# A GIS-driven integrated real-time surveillance pilot system for national West Nile virus dead bird surveillance in Canada

**DOI:** 10.1186/1476-072X-5-17

**Published:** 2006-04-20

**Authors:** Jiangping Shuai, Peter Buck, Paul Sockett, Jeff Aramini, Frank Pollari

**Affiliations:** 1 Foodborne, Waterborne and Zoonotic Infections Division, Centre for Infectious Disease Prevention and Control, Public Health Agency of Canada, Tunney's Pasture, Ottawa, Canada

## Abstract

**Background:**

An extensive West Nile virus surveillance program of dead birds, mosquitoes, horses, and human infection has been launched as a result of West Nile virus first being reported in Canada in 2001. Some desktop and web GIS have been applied to West Nile virus dead bird surveillance. There have been urgent needs for a comprehensive GIS services and real-time surveillance.

**Results:**

A pilot system was developed to integrate real-time surveillance, real-time GIS, and Open GIS technology in order to enhance West Nile virus dead bird surveillance in Canada.

Driven and linked by the newly developed real-time web GIS technology, this integrated real-time surveillance system includes conventional real-time web-based surveillance components, integrated real-time GIS components, and integrated Open GIS components. The pilot system identified the major GIS functions and capacities that may be important to public health surveillance. The six web GIS clients provide a wide range of GIS tools for public health surveillance. The pilot system has been serving Canadian national West Nile virus dead bird surveillance since 2005 and is adaptable to serve other disease surveillance.

**Conclusion:**

This pilot system has streamlined, enriched and enhanced national West Nile virus dead bird surveillance in Canada, improved productivity, and reduced operation cost. Its real-time GIS technology, static map technology, WMS integration, and its integration with non-GIS real-time surveillance system made this pilot system unique in surveillance and public health GIS.

## Background

### West Nile virus spread in North American and Canada

West Nile virus was first isolated in 1937 in the West Nile district of Uganda. Since then, Egypt, Israel, South Africa, and in parts of Europe, Asia and North America have reported West Nile virus infections. In North America, West Nile virus was first reported in New York City in 1999. During 2002, more than 4,000 people in North America became ill after being infected with West Nile virus. The latter was the largest outbreak of West Nile virus infection recorded. West Nile virus activities were reported in more than 40 states in the United States in 2004 [1].

In August 2001, West Nile virus activity was first reported in Canada, when the virus was found in dead birds and mosquito pools in Southern Ontario. In 2002, Canada reported its' first confirmed human cases in parts of Quebec and Ontario. The virus was also found in birds, horses or mosquitoes in Nova Scotia, Quebec, Ontario, Manitoba and Saskatchewan. Two people in Alberta were infected, but thought to be travel-related [1]. In 2005, five provinces (Quebec, Ontario, Manitoba, Saskatchewan and Alberta) have reported West Nile virus activities in Canada, and confirmed cases of human infection were reported in Ontario, Quebec, Saskatchewan, and Alberta [2].

In addition to its' public health impact, West Nile virus has also become an economic burden. In Louisiana, U.S, the total cost of 2002 West Nile virus epidemic was estimated at $20.1 million: 54.22% for illness and 45.78% for public health response [3].

From 1999 to 2003, West Nile virus has spread from New York State to California in US [4]. In Canada, the same East-to-West spread has brought concerns. In early 20005, British Columbia Centre for Disease Control predicted that West Nile virus would appear in British Columbia in 2005 [5]. West Nile virus has become a serious public health threat in Canada. In 2003, there were 1,478 clinical cases of human infection, with 12 deaths. In 2005, there were 224 clinical cases of human infection, with 12 deaths [2,6]. Ontario, Quebec, Manitoba, Saskatchewan, and Alberta have reported positive dead birds. West Nile virus has not spread to British Columbia. In this situation, a national West Nile virus surveillance is critical for West Nile virus risk protection, prevention and control.

### National West Nile virus surveillance practice in Canada

To protect the health of Canadians, and to monitor and control West Nile virus in Canada, the Public Health Agency of Canada has developed corresponding surveillance systems and infrastructure for West Nile virus surveillance in the past, and has been constantly enhancing them.

Public Health Agency of Canada organized a national steering committee for West Nile virus surveillance in February 2000. This committee includes Public Health Agency of Canada, Health Canada, Provincial Ministries of Health, Conservation, Natural Resources, the Department of National Defence, Environment Canada, the Canadian Food Inspection Agency and the Canadian Cooperative Wildlife Health Centre. This committee helps plan and coordinate national surveillance, prevention, control and communications activities across federal, provincial and municipal jurisdictions. The Public Health Agency of Canada also works in collaboration with blood services and other interested organizations [7].

In Canada, current surveillance activities focus on birds, horses, mosquitoes and humans. Surveillance is conducted to detect the presence of the virus as early as possible in any given area so that communities can take steps to reduce their risk.

Dead birds will be tested for West Nile virus from late April until the first hard frost. The tests are done mainly on crows, jays, magpies and ravens as they seem to be the best indicator for determining whether people in particular areas are at risk [8-12]. Mosquito surveillance focuses on establishing the count of mosquito species in a given area through mosquito collection and testing. Mosquito surveillance helps to identify how different species spread the virus to birds, animals and people, and determines the best intervention to reduce the risk of infection. In human surveillance, health care providers are responsible for identifying symptoms of West Nile virus infection in patients and requesting laboratory tests, where appropriate. Consequently, they report all probable and confirmed cases of West Nile virus infection to their corresponding health authorities [7]. West Nile virus in horses is also monitored.

Foodborne, Waterborne and Zoonotic Infections Division (FWZID) coordinates the national response to West Nile virus (with multiple federal and provincial governmental and non-governmental agencies) through coordination and collaboration, national surveillance and data management, document development, communications, and research activities. The division takes a proactive approach to West Nile virus risk management activities.

Since 2001, FWZID has established a West Nile virus surveillance web site to provide comprehensive information related to the West Nile virus threat, protection, and public education. The web site consists of both a public and a restricted access section. The public site provides background and current information on the latest surveillance findings and scientific knowledge about the risks associated with the West Nile virus as well as links to other relevant websites. The restricted section provides a quick access to key documents, current surveillance data and other relevant information for the various jurisdictions involved in the issue. It also provides a venue for sharing draft documents including communication materials.

Several provinces have also developed GIS capacities in their West Nile virus surveillance. Such as the British Columbia Centre for Disease Control has developed an interactive mapping web site related to West Nile virus [5]. In Quebec, Institut national de santé publique du Québec developed an "Integrated System for Public Health Monitoring of West Nile Virus" [13]. This is an internal system, using JMap as the mapping engine, and Microsoft SQL Server as the database engine.

### The role of West Nile virus dead birds surveillance

In West Nile virus surveillance, it is costly and resource-intensive to test all the dead birds, mosquitoes, horses, and humans. In order to reduce resource consumption, a comprehensive surveillance could be conducted on selected species.

In a study, Yaremych et al has found that 90.5% of the dead American Crows were confirmed positive for West Nile virus during the months of May through October in Illinois, 2002 [12]. In New York State, Bernard et al found that among the 1,687 dead American Crows, 47% of them were tested positive for West Nile virus [8]. Watson et al found a spatial association between early-season crow deaths and human West Nile virus infection cases [11]. Mostashari et al reported that dead bird data could be used for early warning of West Nile virus activity in small areas. Through spatial-temporal cluster analysis of dead bird data, jurisdictions could develop intensified early larval control programs and prioritize regions for surveillance [9].

Liu et al developed a mathematical model to predict West Nile virus activity. The model indicated that dead bird was a sensitive indicator for detecting West Nile virus activity at the national level. The basic production number indicated that even if there was no mosquito or other surveillance data, the dead bird surveillance data would still reveal the prevalence of West Nile virus and its spatial spread [10].

This scientific evidence suggest that dead bird surveillance can provide scientific information to understand West Nile virus activities so other surveillance activities might be simplified, which will help control surveillance and sample testing cost. These evidences also suggest that dead bird surveillance can provide information to monitor the spread of West Nile virus over space, and can be used to prioritize geographic regions for mosquito control interventions [11].

### The role of GIS in public health surveillance

GIS has been widely used in public health. Funded by World Health Organization, United Nations Children's Fund, US Agency for International Development and individual United Nations member states, many countries have established a network of resources and expertise in GIS development for public health. World Health Organization established a Geographic Information System for Leprosy Elimination [14]. World Health Organization also developed other GIS systems for public health mapping [15]. The US Centers for Disease Control and Prevention developed a GIS and public health web site [16].

GIS has been widely applied in different aspects of public health surveillance in Canada. The Public Health Agency of Canada developed a *Disease Surveillance On-Line *web site that provides mapping and other services for cancer, cardiovascular diseases, major chronic diseases, notifiable diseases, and injury surveillance [17].

GIS also played an important role in early West Nile virus surveillance systems. A web site provides interactive mapping [18]. There is also a static map web site to display English and French map images for five regions in Canada. These maps and map data are updated on a daily basis. An ArcView application was developed to process the daily surveillance data, to produce daily data for interactive maps, and to generate static maps. The well-received GIS services in West Nile virus surveillance demonstrates that GIS could enrich and enhance public health surveillance.

### Need for a GIS-driven integrated real-time West Nile virus dead bird surveillance system

Since 2001, two desktop applications and four web systems have been developed to accommodate the changing needs of national West Nile virus dead bird surveillance. These systems are operated in different locations. The operation cost is high because these applications and systems have to be maintained and operated manually. There is also a delay between data reported to the database and data available for web mapping and public access. The evolution of those applications and system demonstrated the urgent needs for public health professionals, policy-makers, and various end-user groups to have an integrated surveillance system for real-time West Nile virus data collection and management, and to monitor, track, and understand public health events in a spatial context.

The need for GIS in national West Nile virus dead bird surveillance arose from 2001, and the format of GIS applications has been quickly evolving. In 2001, a static map web site was developed, ArcView was used to create professional maps and to export them as images. The image maps were posted on the static web site. In 2002, HTML ImageMapper was introduced to transform ArcView maps and data into interactive maps on a new interactive web map site. In 2003, a new interactive map web site was developed using MapServer [19]. A new automated ArcView application was developed to produce daily summary data for this interactive map web site and to create 10 static maps daily. This new MapServer site and the new ArcView application greatly reduced workload and file size, and were used in 2003 and 2004. The static map web site has been kept since 2001. The major problems were that these GIS application required lots of human resource input, and were separated from database or other web systems. Any change in one application may need changes in several other systems.

Through 2001–2004, new GIS needs for West Nile virus surveillance have been constantly rising. Theses needs included the quality improvements of spatial data through spatial validation, automation of spatial data generation, capacity to allow end users to produce maps for any date periods, addition of security control in incident point display, more methods to generate coordinates using different location information, automation of static map generation, more interactive web mapping functions, integration of the various web mapping systems and desktop GIS applications into a single surveillance system, animation of static maps for pattern recognition, provision of GIS support in data entry and editing, and the integration of GIS components and surveillance components.

The Public Health Agency of Canada and its partners have developed the Canadian Network for Public Health Intelligence (CNPHI). CNPHI "*is targeted at improving the capacity of the Canadian public health system to reduce human illness associated with infectious disease events by supporting intelligence exchange, surveillance activities, and outbreak investigations*." [20]. As the CNPHI initiative to enrich and enhance national West Nile virus surveillance, a GIS-driven comprehensive real-time surveillance pilot system is designed to serve national West Nile virus dead bird surveillance, information sharing, data reporting and GIS services.

Meanwhile, GeoConnections program promoted Open GIS technology in Canadian public health fields. This promotion drew FWZID's attention because it addressed one of the West Nile virus surveillance GIS needs – accessing external geospatial data for risk assessment and decision-making. CNPHI, FWZID and partners applied for a GeoConnections funding opportunity and received funding from GeoConnections to integrate real-time GIS and real-time surveillance with Open GIS web mapping service (WMS) that implements Canadian Geospatial Data Infrastructure (CGDI).

According to the technical and business requirements for national West Nile virus dead bird surveillance, the following features were identified as very important: (1) ability to access WMS data sources; (2) ability to generate WMS services; (3) provide multiple language support; (4) transfer data from provincial databases to national database (need ETL); (5) use Oracle and ArcIMS; (6) integrate internal and public web sites in a single system; (7) generate, store and display daily static maps; (8) use six different geo-referencing methods to generate latitudes and longitudes; (9) allow users to make real-time maps for any user-defined date period; and (10) follow federal government web site development standards.

This pilot system addressed the urgent need for an integrated real-time national West Nile virus dead bird surveillance system. This pilot system was driven by GIS, especially the newly developed real-time web GIS technology, in order to provide various GIS services, and to integrate real-time web GIS and WMS into a web-based real-time surveillance infrastructure.

FWIZD initially planned and designed this pilot system, and invited eight partners from federal, provincial, non-governmental organizations, academic, and private sectors to implement the development of this pilot system in 2004. The two objectives of developing this pilot system are to build a GIS-driven web-based integrated real-time surveillance system in order to streamline and strengthen national West Nile virus dead bird surveillance, and to build WMS capacity in surveillance systems for spatial data sharing through CGDI.

## Results

### System overview

Driven by an innovative real-time web GIS technology, this pilot system is planned, designed and developed as an integrated system to track, update, manage, map, query, and deliver West Nile virus dead bird surveillance data, and serve the public health professionals and the general public with real-time interactive maps and data.

**Figure 1:**
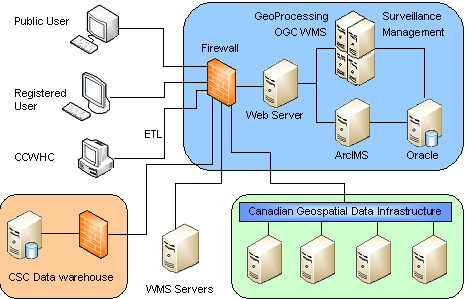
**System structure**. This pilot system allows both public and internal users to access different parts of the system. Data can be reported through Internal data reporting page, or by a standalone ETL tool if provinces already have surveillance reporting systems. This pilot system can access both open WMS and secure WMS.

This pilot system has four major components: a public web site, an internal web site, service and management tools, and WMS capacities. In addition to having the features of a conventional web-based non-GIS surveillance system, it also has comprehensive real-time web GIS capacities and WMS capacities. Figure 1 shows the structure and components of this pilot system.

**Figure 2:**
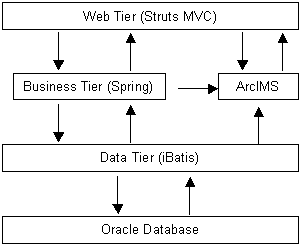
**System architecture**. This pilot system uses Structs, Spring, iBatis as the frameworks for development. These frameworks have enhanced the system. It uses Oracle and ArcIMS to manage data and web GIS.

This pilot system is a J2EE application. The application's functionality is separated across different tiers, or functional layers, to provide separation of responsibilities. This architecture provides many advantages, such as reusability, improved scalability, full Model-View-Controller (MVC) separation, easy maintenance, separation of business logic from presentation logic, a consistent library of reusable code, a standard validation framework, and a standard internationalization framework for multiple language support. Figure 2 shows the architecture of this pilot system.

The web tier uses the Struts framework based on the Model 2 approach, a variation of the classic MVC design paradigm. The core of the Struts framework is a flexible control layer based on standard technologies like Java Servlets, JavaBeans, ResourceBundles, and XML, as well as various Jakarta Commons packages [21]. Struts creates a safe development and integration environment and solid tools for developers, and makes many of problematic areas of web development faster to develop and easier to maintain. The business tier is based on the Sprint framework to manage business objects because it can effectively organize the middle tier objects. Spring framework takes care of plumbing and therefore helps reduce development and maintenance efforts. The iBatis Data Mapper framework simplifies the interaction of database and Java applications. iBatis couples objects with stored procedures or SQL statements using a XML descriptor. The major advantage of the iBatis Data Mapper over object relational mapping tools is its simplicity [22].

In order to protect data confidentiality and also differentiate the functionalities between general public users and registered internal users, this pilot system developed a public-internal infrastructure. It divides the system into a public web site and an internal web site. In the public site, users can access aggregated, embargoed information. Users can produce maps for any date period. Users can do many GIS operations on the real-time map, such as "Identify", "Select by Rectangle", and display the spatial query results. The internal web site has more functions and services. In addition to all the functions available in the public web site, the internal web site also provides surveillance data reporting and editing, GIS support for data reporting and editing, spatial query, six different geo-referencing methods, point data mapping, remote data transfer, system management, and security control.

This pilot system utilizes two commercial software packages for web GIS and data management: ArcIMS (version 4.0.1 or above) and Oracle (version 9 or above). It also uses two open-source software packages: Apache and Tomcat. These two open-source software packages provide stable support for an ArcIMS production system. The architecture of this pilot system is designed in a way that the web server, the ArcIMS server, and the database server can be on the same or different server boxes.

### Real-time surveillance components

Public health surveillance requires the acquisition and analysis of health event data. The data can be used for retrospective analysis to understand the different aspects of public health events and their relationship with social, economic, and environmental factors. The data can also be used for early warning analysis. All these analyses require consistent and high-quality data. The early warning analysis also requires a real-time data acquisition within a surveillance system. This pilot system developed a real-time surveillance data reporting and editing component to support real-time data acquisition, to provide the capacities to validate data, and to link to real-time GIS. This real-time surveillance component is an internal function, and can only be accessed by registered users.

**Figure 3:**
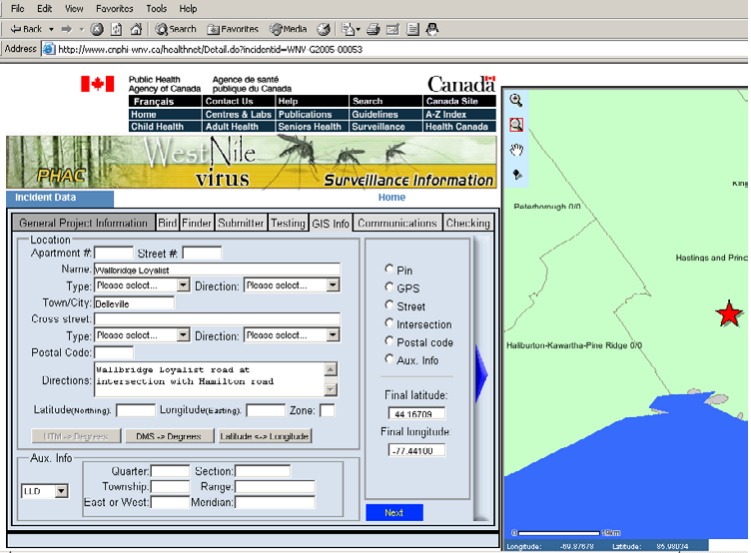
**Internal data edit**. This pilot system deals with over 100 fields for surveillance data reporting and editing. Specific functions have been developed to integrate GIS capacity and surveillance data reporting/editing. A map is present to assist spatial data element input and verification.

As a key capacity of this real-time surveillance pilot system, the data reporting web page and data editing web page provide support for accurate data collection and quality control. These two web pages handle over 100 data fields, and many data validation rules are implemented. Figure 3 shows the interface of the data reporting page. The data elements in these two web pages are divided into eight sections. One of the sections is the GIS information section that contains data elements for identifying the location of a reported dead bird. Both data reporting and data editing processes are supported by the real-time GIS capacity. Seven types of location information can be reported and used for generating geographic coordinates.

The data reporting web page and data editing web page are almost identical apart from the fact that the data editing web page has pre-filled data from the database for each field while the data reporting web page does not. Many pick lists are provided to maintain data integrity and eliminate data input errors. Since validation during data entry or editing is important for quality data improvement, this pilot system uses Struts validation framework to implement comprehensive data validation.

To facilitate data editing, users can search records by providing incident ID, testing result, date period, completeness of spatial data, and the valid status of spatial data elements. The search results can be sorted by the headers of the result table. A number of buttons are provided for users to easily navigate through the results. Clicking any row of records will open the data editing page that is filled with the corresponding data.

**Figure 4:**
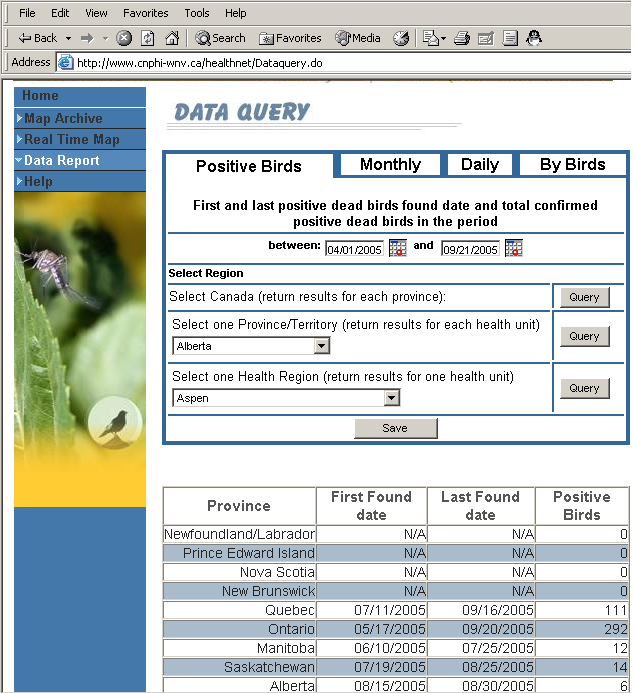
**Data query**. This pilot system provides four types of queries at three spatial levels. Users can query data at different period (including real-time data and historical data). Data can be saved as well.

To assist surveillance data analysis, this pilot system provides four types of queries: (1) earliest and latest dates that the confirmed positive birds were found for a period; (2) monthly statistical summary for a specific month; (3) statistical information for a specific day; and (4) statistical information by bird types for a period. These queries can be operated at three spatial levels: national, provincial, and health unit. Users can query data at different period (including real-time data and historical data). Data can be saved as text file. Figure 4 shows the query interface for querying real-time and historic data. To optimize system performance, this page uses dynamic dependent list boxes. Uses can change provinces and the corresponding health units will be quickly populated without communicating with the server.

### Real-time web GIS components

The most significant contributions of this pilot to our West Nile virus surveillance system are the identification, development and integration of (a) a new real-time web GIS technology based on ArcIMS, and (b) a number of web GIS functions and capacities that are important to public health surveillance. In this pilot system, real-time GIS technologies and capacities are much more comprehensive than just producing web maps. Table 1 lists the major GIS functions of this pilot system. In this pilot system, web GIS is designed as a bridge to link surveillance components, GIS components, management components, and spatial operations systematically, and form a GIS-driven integrated real-time public health surveillance system.

**Table 1:** Major GIS functions of this pilot system

Major GIS Functions	Public Site	Internal site
1. Real-time map page		

• Aggregated map		
• Embargo implementation		
• Fixed zoom, interactive zoom		
• Polygon feature identify, select by rectangle, clear selection		
• Pan, measure distance, print, save map		
• Fixed legend		
• Zoom to provinces		
• Large map		
• Open, close spatial query results		
• Integrated legend for WMS, real-time data, and ArcIMS layers		
• Bird location point map		
• OGC WMS data access		
• Point feature identify, select by rectangle, clear selection		
• Link point feature to data editing page		

2. Static map page		

• Switch to different regions		
• Animation of static maps		
• Define date period for static map retrieval		
• Embargo		

3. Services		

• Static maps generation		
• Geo-referencing		
• Spatial data validation		
• Incident point shapefile generation		
• WMS update		

4. Data reporting and editing pages		

• Real-time map		
• Interactive zoom		
• Spatial validation result display		
• Retrieve mouse point coordinate		

**Figure 5:**
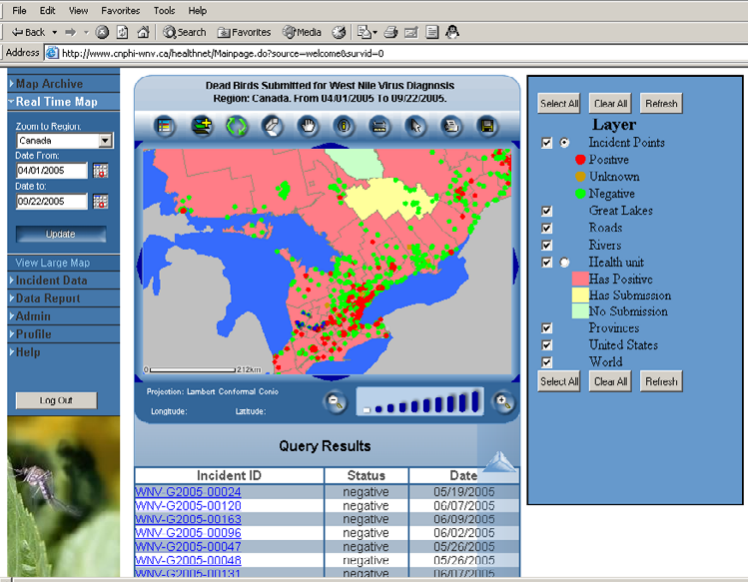
**Internal real-time map**. The internal real-time map provides aggregated map at health unit level, bird location mapping, and OGC WMS maps. The legend allows users to query real-time data at health unit level and the bird points. Users can click the incident from the query result table and switch to data editing page.

In this pilot system, the definition of real-time GIS is also unique. While many real-time web GIS systems provide maps reflecting the most recent information only, this pilot system allows users to generate maps for any date period. This may be important for public health surveillance and outbreak response, for analysis, and for decision-making support. For example, if users need to view situations in a specific week or weeks, month or months, they can set the start date and end date, and submit the request; a new map will be generated. If the database has historical data, the system can generate historical maps as well. These real-time maps are interactive. All the maps support "identify" and "select by polygon" spatial query functions. An integrated dynamic legend is provided for the internal real-time map (Figure 5), and a simple legend is provided for the real-time public map.

Real-time GIS is also integrated with data reporting and data editing. Users can select incidents from the real-time map. The selected points are highlighted, and the selection results are listed in the query table below the map. A hyper link is created for each incident in these selection results. If the user clicks on the ID of an incident, the real-time map page will be switched to the data editing page. The specific incident is also highlighted in the map within the data editing page (Figure 3).

To support real-time web GIS and mapping, this pilot system takes a non-shapefile approach. All surveillance data are stored in an Oracle database system. The spatial relationship between surveillance data and maps are generated on the fly, and are cleaned after the session is timed out. The ArcIMS map services only provide reference maps. Simply accessing the ArcIMS map service will not access the surveillance data or map: this is efficiently protects data confidentiality without having to develop intensive security control components. This approach also largely simplifies the group access management for the internal real-time map. In the internal real-time map, users are able to view and query bird by location. Controlled by this mechanism, users only have access to the bird point data within their group permission. For example, a provincial user only sees the bird points within his or her province or territory on the map.

An important requirement for this pilot system is to improve the quality of spatial data using the real-time GIS capabilities. The system generates and validates coordinates using six different geo-referencing methods to generate latitudes and longitudes: on-screen pinpoint, GPS results reporting, address geocoding and address intersection geocoding, postal code lookup, map grid (for Nova Scotia), and legal land description (for Alberta, Saskatchewan, and Manitoba).

Based on the analysis of the quality and problems of historical data, a number of data quality issues have been identified. Those issues have to be resolved. Otherwise, the real-time map and real-time query will generate incorrect outputs. As the solution, a spatial validation process has been developed. The incident records are validated immediately after the records are reported to or edited in the database. Two types of spatial validations are conducted. One is spatial relation validation; the other is administrative boundary validation. The two classifications, criteria, and usages are listed in Table 2 and Table 3.

**Table 2:** Incident record classification for spatial relation validation

Classification	Validation Criteria	Usage
Complete	Has correct geographic coordinates, the coordinates fall in the correct health units, and the reported health units fall in correct provinces or territories.	Data can be used for the aggregated mapping and bird location point mapping.
Not complete	The reported health units fall in correct provinces or territories. But there are no geographic coordinates, or the coordinates are incorrect.	If there are no coordinates, the bird location point cannot be mapped; Data is flagged for internal users to provide correct coordinate information.

**Table 3:** Incident record classification for administrative boundary validation

Classification	Validation Criteria	Usage
Valid	Reported health units fall in the reported provinces or territories.	The record is counted in all process of the system.
Not valid	Reported health units do not fall in the reported provinces or territories. This will cause confusion in statistics, because the total number summarized by health units will be different from total number summarized by provinces or territories.	These invalid records are stored in the database. However, the map and query processes will not count them. Data is flagged for internal users to edit.

A unique static map component has also been developed to create professional static maps. All the static maps are stored in an Oracle database, and can be served as a digital atlas of historical West Nile virus activities in dead birds. During the surveillance season, twenty static maps are generated each day for the country and four regions, and for internal and public users. Each of the five regions has an English version map and a French version map. The national map does not have labels for health units, however, the four regional maps have labels showing health unit name, the number of confirmed positive birds, and the number of tested birds. Retrieving these maps does not require ArcIMS support. This approach optimizes system performance, and reduces the overhead of ArcIMS server. The map archive page allows users to define the date period, to retrieve the static maps from an Oracle database, and to animate the static maps. The animation will help visualize how West Nile virus spreads over space in the given period.

### WMS capacity integration

Another important enrichment of this pilot to previous West Nile virus surveillance system is the integration of WMS capacities. Within the internal web site, capacities have been developed to retrieve various WMS data sources, and can overlay the retrieved WMS layers on top of the real-time map. Users are able to select a WMS server and an available WMS layers via two dropdown boxes. The WMS data layers can be landscape, land use, temperature, vegetation, or other environmental factors. Putting these WMS layers together with the real-time surveillance map helps to visualize the spatial relationship between these potential determinants and the West Nile virus activity. The WMS capacities can be used for decision-making support, public education on West Nile virus protection (such as providing information showing the association between hot spots and wetland).

The primary objectives of developing the WMS capacities are to access WMS spatial data in CGDI nodes, and to make extensive use of internal secure data, such as the WMS spatial data in the internal secure spatial data warehouse. In addition, this pilot system can also access other publicly available WMS services through the Internet.

**Figure 6:**
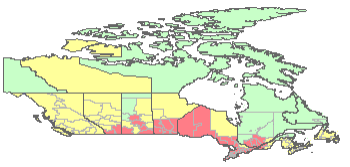
**Sample WMS map**. This pilot system provides WMS and makes it publicly available. It can be accessed through standard OGC GetMap request (i.e. ).

This pilot system also provides its own national West Nile virus dead bird surveillance WMS service to the public. This WMS service is updated daily using the real-time data in the Oracle database and reflects the most recent surveillance results. Users can acquire this WMS layer, and overlay it on their own data for analysis and visualization. Figure 6 shows an example of the WMS map.

### Data transfer capacity

In the national West Nile virus dead bird surveillance process, different stakeholders may collect data at the provincial level, and then report to the federal database system. In some cases, provincial partners may have to enter data to their own system first, and enter the same data into the federal system again. This practice increases operation costs and may also lead to the inconsistency of data between provincial and federal database systems due to the repeated data entry. A tool is needed to transfer provincial data to the federal surveillance system automatically, frequently, and consistently.

In addition, the dead bird surveillance data was manually downloaded in the old systems. The downloaded data was imported into the ArcView application for GIS process. A daily data set was produced for the interactive web map, and five English and five French static maps were also produced. These processes could not support real-time surveillance because there was a delay between the time when the surveillance incident data was entered into the database and the time when the corresponding interactive maps and static maps were available on the web sites. To address these issues, this pilot system developed a data extract, transformation, and loading (ETL) tool. This tool has an ETL server component residing on a server of the pilot system, and an ETL client component residing on the data provider's computer. The data providers can upload and/or download data based on their credentials. The data for upload has to been prepared following a predefined format using a specific delimiter and an order of fields. To maximize the performance of the system, only new or modified records are transferred to the pilot system each time. When the upload is finished, certain data elements are also downloaded from the database, deepening on the credentials of the users.

The advantage of this ETL is that clients do not have to invest on any special software or specific platforms. The ETL client is a Java application. The client user only needs to install the free J2SDK1.4 on their computer, and has Internet connection. This is very useful and convenient for users in different networks and platforms. The ETL client usually runs as a scheduled task.

### Background services

To support the integration of real-time GIS, real-time surveillance, and WMS, four server-side background services run on the server. They either support some functions of the web pages, or provide additional services for national West Nile virus surveillance that are not available from the web pages.

The static map generation service constantly runs on the server using a Java application. This service extracts the real-time data from the Oracle database system, and generates map images based on those data. The images will then be stored in the Oracle database system. These map images contain many map elements, such as a title, date of creation, map inserts, logo, legend, labels, and statistical data table. Administrators can change the schedule of static map generation from a web page.

The second service is the point shapefile production process. This service extracts the real-time data from the Oracle database system, and generates fourteen point shapefiles for the country and for each of the thirteen provinces/territories. These data are emailed to registered users. The third service is used to extract the real-time data from the Oracle database system and update the West Nile virus dead bird WMS data source. The last service is to build an EXCEL file using the real-time data in the Oracle database system. The EXCEL file has specific data requirements, and contains national and provincial summaries. It will be converted to daily PDF reports. The schedules of these three services are managed through Windows scheduled tasks.

### System management

The pilot system has also developed a number of web-based management tools. The administrators use these tools to manage the different parts of this pilot system:

• System configuration tool: provide configuration information for the system.

• Schedule management tool: set, edit, or delete a task schedule. The tasks can be set to execute once, daily, weekly, or monthly.

• Group management tool: create, manage, edit, and delete groups. Each group is granted access to the data of one or several provinces/territories for West Nile virus surveillance activities.

• User management tool: create, manage, edit, and delete users. Each user belongs to one or several groups.

• Session control tool: monitor and control user sessions. Administrator can end a session (such as for troubleshooting). If a session is ended, the corresponding user will get a session timeout error. If the user wants to continue accessing the site, the user will have to start a new session (e.g. open a new browser, login again).

• Embargo management tool: set the embargo days. At the request of the Public Health Agency of Canada's provincial/territorial partners, a two-business-day embargo is placed on dead bird positive testing results. This allows the provinces or territories to notify all affected areas and personnel before the tested information is posted on the Agency's West Nile virus web site [1].

• WMS services management tool: register, edit, delete, activate or de-activate WMS. Once a WMS is registered in this pilot system and is made active, internal users will be able to access the corresponding WMS layers.

### Web GIS clients for public health

**Figure 7:**
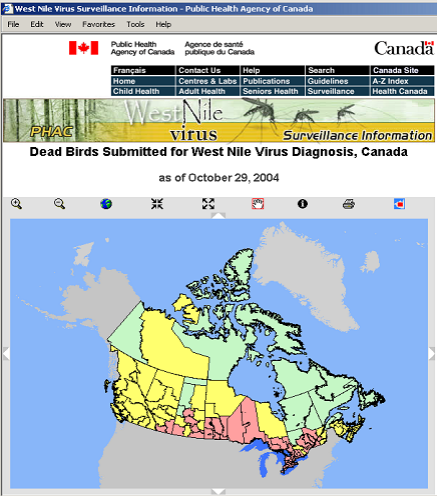
**Lightweight mapping client**. This web mapping client provides an interactive mapping client that is much lighter than the out-of-box ArcIMS HTML viewer. This client does not use frame, therefore can be easily integrated into other web systems. This client is easy to customize, and has a good look-and-feel.

Based on the technology developed in this pilot system, six different types of web GIS clients become available for public health surveillance. These web GIS clients can provide a wide range of web GIS services to public health in a variety of formats and meet a variety of functionality, management, resources, and financial requirements. The six web GIS clients are:

1. Public-internal dual client: this is the full system of this pilot system. It consists of a public client and an internal client. It has all the components of internal system, public web site, background services, and system management.

2. Internal real-time surveillance client: derived from the full system, this client provides a web system for an internal surveillance. It has all the web pages, services, and tools, but it does not provide a public web site. This client is useful for organizations that only need an internal surveillance system.

3. Public real-time mapping and data query client: derived from the full system, this client provides a public web system which has a real-time mapping page, a static map page, and a surveillance data query page. It is useful for organizations that already have internal or traditional non-GIS surveillance systems, but needs web GIS services.

4. Lightweight mapping client: this client is an interactive mapping client that is much lighter than the out-of-box ArcIMS HTML viewer. This client does not use frame, therefore can be easily integrated into other web systems. This client provides interactive mapping support for existing spatial data as does the out-of-box ArcIMS HTML viewer, but is relatively easier to customize, and has an improved look-and-feel (Figure 7).

5. WMS-integrated mapping client: this client is an enhancement to the out-of-box ArcIMS HTML viewer. WMS capacity has been developed and added to the ArcIMS HTML viewer, so users can easily retrieve and display WMS layers. This client also supports the WMS GetCapabilities request.

6. Multi-purpose mapping centre: this client is an enhancement to the out-of-box ArcIMS HTML viewer. Through this single application, users can access all the available map services on the same ArcIMS server. This can be convenient for end users, GIS developers, and system administrators.

### System implementation and future plan

This pilot system has been deployed to production in 2005, and has successfully served the 2005 national West Nile virus dead bird surveillance [23]. This pilot system has significantly streamlined and enhanced national West Nile virus dead bird surveillance, because this single system replaced the six different previous systems. It has also eliminated the needs to manually process data or maps, so the resources needs and operation cost have been reduced significantly. It has also eliminated the need to transfer data among different locations manually.

**Figure 8:**
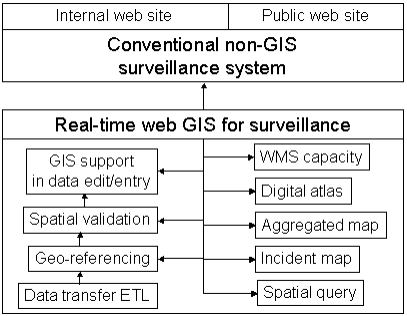
**Enrichment and enhancement to conventional surveillance system**. This pilot system developed a number of web GIS components to enrich and enhance conventional surveillance systems that do not have GIS components. These components provide new services such as digital atlas using static maps, real-time mapping, comprehensive geo-referencing, and WMS, spatial data validation.

This pilot system enriched and enhanced conventional surveillance systems that do not have GIS components, because it integrated traditional web-based surveillance, real-time web GIS, and WMS (Figure 8). The enriched features include real-time web GIS technology and services, GIS and mapping support in data entry and editing, static map technology, non-shapefile approach for real-time mapping, and WMS capacities. The enhancements include spatial data quality enhancement through spatial data validation, embargo implementation, internal and public infrastructure, multi-lingual capacity, a data transfer tool, database-based GIS data security management, and service automation.

It is planned that this pilot system will be used to server other zoonotic disease surveillance in the future. One of the major initiatives to implement this plan is a proposal to a new GeoConnections funding opportunity. FWZID, CNPHI and collaborators have submitted a proposal for the 2006 GeoConnections funding opportunity in early 2006. The proposal has been accepted, and an agreement will be finalized in April 2006. One of the objectives of this proposal is to extend this pilot system to serve other national zoonotic disease surveillance, including avian influenza and Lyme disease. New WMS services and metadata services are to be developed through this proposal. The proposal will also gain improved GIS functionality, and will greatly leverage the benefits of CGDI integration through the chaining of CGDI resources to enhance national zoonotic disease surveillance. The proposal, building on the successful GIS technologies, successful management, and large user groups of CNPHI and this pilot system, will produce a GIS-driven, integrated, CGDI-compliant, real-time national zoonotic disease surveillance system.

## Conclusion

This paper presents a real-time GIS-driven surveillance pilot system, its context, its architecture and components, its various GIS functionalities, and examples of its outputs. This pilot system has been developed to address the urgent needs for an effective, GIS-enabled, WMS-enabled, integrated real-time surveillance system for national West Nile virus dead bird surveillance. This pilot system allows users to track and monitor national West Nile virus dead bird surveillance information in real-time. The real-time surveillance and real-time GIS capacities have enhanced data reporting and editing and reduce the delay of surveillance information. The real-time GIS capacities have addressed the urgent GIS needs in national West Nile virus surveillance. The pilot system has also identified the major GIS functions and capacities that are important to public health surveillance. The non-shapefile approach can effectively protect data confidentiality, which is very important in public health surveillance. The public-internal infrastructure supports the different requirements of the general public and public health professionals in a single system. The WMS capacities allow users to retrieve WMS layers and put on the real-time surveillance map in order to analyze the spatial association between incidents and environmental factors. The six web GIS clients provide a wide range of GIS tools for public health surveillance.

This pilot system has enriched and enhanced national West Nile virus dead bird surveillance in Canada, improved productivity, and reduced operation cost. Its real-time GIS technology, static map technology, WMS integration, and its integration with conventional web-based real-time surveillance system of this pilot system demonstrate a unique approach in the design and development of public health surveillance systems.

## Abbreviations

CGDI: Canadian GeoSpatial Data Infrastructure

CNPHI: Canadian Network for Public Health Intelligence

ETL: Data extract, transformation, and loading

FWZID: Foodborne, Waterborne and Zoonotic Infections Division, Public Health Agency of Canada

GIS: Geographic information system

MVC: Model-View-Controller framework

OGC: Open Geospatial Consortium Inc.

WMS: OGC web mapping service

## Authors' contributions

JS led the development of this pilot system, and conceived and drafted this manuscript. PB, PS, JA, and FP participated in the management and development of the system, and assisted the integration of this pilot with CNPHI. All authors read and approved the final manuscript.

## References

[B1] Public Health Agency of Canada (2005). West Nile virus – History. http://www.phac-aspc.gc.ca/wn-no/index_e.html.

[B2] Public Health Agency of Canada (2005). Human Results – 2005 Program: West Nile Virus Neurological Syndromes, West Nile Virus Non-Neurological Syndrome and West Nile Virus Asymptomatic Infection, Diagnosis by Health Region, Canada as of September 17, 2005. http://dsol-smed.phac-aspc.gc.ca/wnv3/map_e.phtml?appname=human.

[B3] Zohrabian A, Meltzer M, Ratard R, Billah K, Molinari NA, Roy K, Scott II RD, Petersen L (2004). West Nile virus Economic Impact, Louisiana, 2002. Emerging Infectious Diseases.

[B4] Reisen W, Lothrop H, Chiles R, Madon M, Cossen C, Woods L, Husted S, Kramer V, Edman J (2004). West Nile virus in California. Emerging Infectious Diseases.

[B5] British Columbia Centre for Disease Control (2006). Interactive GIS Mapping for West Nile Virus. http://maps.bccdc.org.

[B6] Public Health Agency of Canada (2003). Human Results – 2003 Program: West Nile Virus Neurological Syndromes, West Nile Virus Fever and West Nile Virus Asymptomatic Infection Diagnosis by Health Region, Canada as of December 31, 2003. http://dsol-smed.phac-aspc.gc.ca/wnv3/map_e.phtml?appname=human&season=2003.

[B7] Public Health Agency of Canada (2005). Surveillance, Education, Prevention and Response. http://www.phac-aspc.gc.ca/wn-no/surveillance_e.html.

[B8] Bernard KA, Maffei JG, Jones SA, Kauffman EB, Ebel GD, Dupuis II AP, Ngo KA, Nicholas DC, Young DM, Shi PY, Kulasekera VL, Eidson M, White DJ, Stone WB, Kramer LD, NY State West Nile Virus Surveillance Team (2001). West Nile virus infection in birds and mosquitoes, New York State, 2000. Emerging Infectious Diseases.

[B9] Mostashari F, Kulldorff M, Hartman JJ, Miller JR, Kulasekera V (2003). Dead bird Cluster as an Early Warning System for West Nile Virus Activity. Emerging Infectious Diseases.

[B10] Liu R, Shuai J, Wu J, Zhu H (2006). Modelling Spatial Spread of West Nile Virus and Impact of Directional Dispersal of Birds. Mathematical BioSciences and Engineering.

[B11] Watson JT, Jones RC, Gibbs K, Paul W (2004). Dead Crow Reports and Locations of Human West Nile virus cases, Chicago, 2002. Emerging Infectious Diseases.

[B12] Yaremych SA, Warner RE, Mankin PC, Brawn JD, Raim A, Novak R (2005). West Nile virus and high death rate in American Crow. Emerging Infectious Diseases.

[B13] Gosselin P, Lebel G, Rivest S, Douville-Fradet M (2005). The Integrated System for Public Health Monitoring of West Nile Virus (ISPHM-WNV): a real-time GIS for surveillance and decision-making. International Journal of Health Geographics.

[B14] World Health Organization (2005). A Geographic Information System for Leprosy Elimination. http://www.who.int/lep/Monitoring_and_Evaluation/gis.htm.

[B15] World Health Organization (2005). Public Health Mapping and GIS. http://www.who.int/health_mapping/en.

[B16] Centers for Disease Control and Prevention (2006). GIS and Public Health. http://www.cdc.gov/nchs/gis.htm.

[B17] Public Health Agency of Canada (2005). Disease Surveillance On-Line. http://www.phac-aspc.gc.ca/dsol-smed.

[B18] Public Health Agency of Canada (2005). Dead Birds Submitted for West Nile Virus Diagnosis by Health Region Canada as of October 29, 2004. http://dsol-smed.phac-aspc.gc.ca/wnv/map600_e.phtml.

[B19] Public Health Agency of Canada (2005). West Nile virus MONITOR. http://www.phac-aspc.gc.ca/wnv-vwn/mon_maps_e.html.

[B20] Research and Technology Development (2006). Canadian Network for Public Health Intelligence. http://www.crti.drdc-rddc.gc.ca/en/investments/research_development/02_0035rd.asp.

[B21] Apache Software Foundation (2005). Struts. http://struts.apache.org.

[B22] Apache Software Foundation (2005). iBATIS. http://ibatis.apache.org.

[B23] Public Health Agency of Canada (2005). West Nile virus Real-time Surveillance. http://www.cnphi-wnvc.ca/healthnet.

